# Conductive Nanofibers-Enhanced Microfluidic Device for the Efficient Capture and Electrical Stimulation-Triggered Rapid Release of Circulating Tumor Cells

**DOI:** 10.3390/bios13050497

**Published:** 2023-04-25

**Authors:** Yisha Huang, Xilin Li, Jianwen Hou, Zhouying Luo, Guang Yang, Shaobing Zhou

**Affiliations:** 1 Institute of Biomedical Engineering, College of Medicine, Southwest Jiaotong University, Chengdu 610031, China; 2 Key Laboratory of Advanced Technologies of Materials Ministry of Education, School of Materials Science and Engineering, Southwest Jiaotong University, Chengdu 610031, China; 3 School of Life Science and Engineering, Southwest Jiaotong University, Chengdu 610031, China

**Keywords:** circulating tumor cells, microfluidics, conductive nanofibers, electrochemical release, electrospinning

## Abstract

The effective detection and release of circulating tumor cells (CTCs) are of great significance for cancer diagnosis and monitoring. The microfluidic technique has proved to be a promising method for CTCs isolation and subsequent analysis. However, complex micro-geometries or nanostructures were often constructed and functionalized to improve the capture efficiency, which limited the scale-up for high-throughput production and larger-scale clinical applications. Thus, we designed a simple conductive nanofiber chip (CNF-Chip)-embedded microfluidic device with a herringbone microchannel to achieve the efficient and specific capture and electrical stimulation-triggered rapid release of CTCs. Here, the most used epithelial cell adhesion molecule (EpCAM) was selected as the representative biomarker, and the EpCAM-positive cancer cells were mainly studied. Under the effects of the nanointerface formed by the nanofibers with a rough surface and the herringbone-based high-throughput microfluidic mixing, the local topographic interaction between target cells and nanofibrous substrate in the microfluidic was synergistically enhanced, and the capture efficiency for CTCs was further improved (more than 85%). After capture, the sensitive and rapid release of CTCs (release efficiency above 97%) could be conveniently achieved through the cleavage of the gold-sulfur bond by applying a low voltage (−1.2 V). The device was successfully used for the effective isolation of CTCs in clinical blood samples from cancer patients, indicating the great potential of this CNF-Chip-embedded microfluidic device in clinical applications.

## 1. Introduction

Circulating tumor cells (CTCs) are a viable population of cells that shed from the primary tumor and/or metastasis, circulating within the bloodstream and containing genomic, transcriptomic, and proteomic information of the patient’s tumor [1,2]. Clinically, numerous studies have predicted the great potential of CTCs as a potential diagnostic tool for diagnosis, prognostication, and therapy monitoring in different cancer types and stages [3]. However, due to the innate rarity and heterogeneity of CTCs as well as the complexity of the metastasis process, the clinical application of CTCs detection still faces several challenges [4,5,6,7]. Extensive research has been performed to develop effective CTCs enrichment and isolation strategies, as they are the fundamental step for the CTCs analysis. These strategies are mainly based on the biophysical properties such as size [8,9,10], mass [11] and deformability [12], and/or biochemical properties of CTCs, namely unique biomarkers expressed by CTCs, like epithelial cell adhesion molecules (EpCAM), HER2, CD26 and CD44 [13], etc. Compared to biochemical-based methods, the biophysical properties-based approaches may introduce false positive results in clinical CTCs capture and isolation [5].

Among a variety of CTCs capture methods and analytical platforms, cell-scale microfluidics can precisely regulate the microfluid flow, providing an avenue for the manipulation of cells based on their biophysical or biochemical properties. In addition, it needs only a small volume of the sample and can be inexpensive and mass-produced easily [13,14,15]. For diverse microfluidic-based CTC isolation methods, complex structures, like microposts [16], were designed and constructed to regulate the flow streamlines to isolate targeted cells. Though effective CTC capture was achieved, the complex geometry brought challenges to scale up for high-throughput production and larger-scale clinical applications. While the herringbone-based high-throughput microfluidic mixing strategy used surface ridges or herringbones in the wall of the device to disrupt streamlines, generating microvortices and maxing the interactions between CTCs and antibody-coated channel surface [17,18], deriving a series of simple and effective herringbone-based CTC capture devices [19,20,21]. What’s more, nanostructured interfaces have been introduced into the microfluidic devices to further improve the capture efficiency of CTCs, enhancing the local topographic interactions between these nanostructures and the nanoscale components (e.g., micro-villi and filopodia) of the cellular surface, and promoting the cell adhesion, which assisted the capture and isolation of the CTCs [22,23]. Inspired by extracellular matrix, electrospun nanofibers possess high specific surface area and high porosity, which can provide more modification sites for immobilization of CTC affinity molecules [24], and are easy to prepare and perform surface modifications on. Thus, different nanofibers-integrated microfluidic platforms have been developed for the isolation of CTCs [25,26,27].

In addition, for further analysis of the CTCs, the safe and effective release of CTCs needs to be considered. A variety of CTC release methods have been developed, such as enzymatic degradation release [28], temperature-sensitive release [29,30], pH-responsive release [31], etc. Among these, the electrochemical release may provide a more cell-friendly, sensitive and rapid release way, which has been widely confirmed in recent reports [32,33,34].

**Figure 1:**
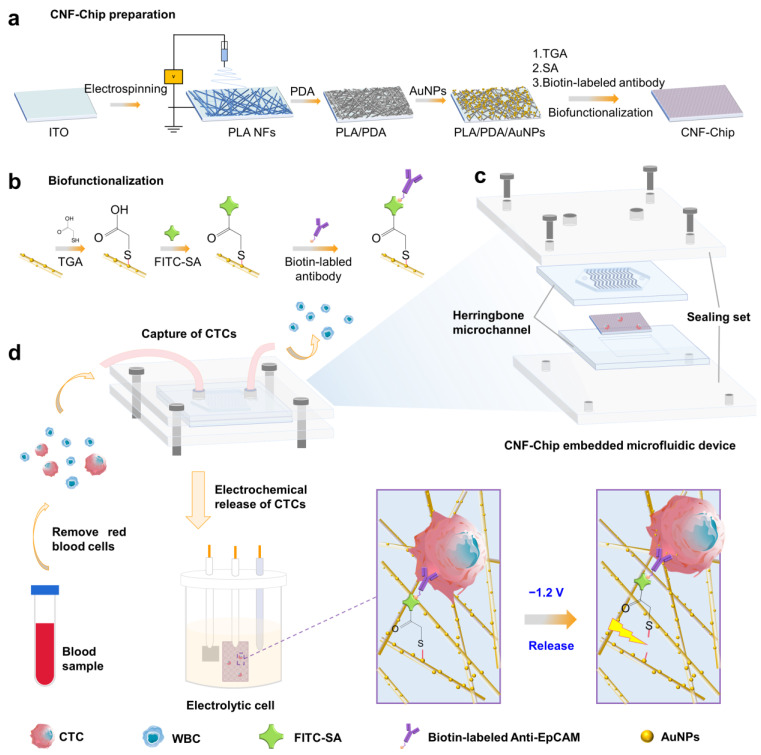
Schematic illustration of preparation and usage of the CNF-Chip-embedded microfluidic device. (**a**) Preparation of CNF-Chip. (**b**) Biofunctionalization of the CNF-Chip. (**c**) Composition of CNF-Chip-embedded microfluidic device. (**d**) Capture and release of CTCs from whole blood by the device.

Therefore, in this work, we developed a facile conductive nanofibers-enhanced microfluidic device for efficient separation and rapid release of EpCAM-positive cancer cells. (Figure 1). The device consists of a conductive nanofiber chip (CNF-Chip), polydimethylsiloxane (PDMS) herringbone microchannel and a sealing set. Specifically, CNF-Chip was prepared by electrospinning polylactic acid (PLA) nanofibers onto an indium tin oxide (ITO) conductive film glass substrate and coating with gold nanoparticles (AuNPs), followed by biofunctionalization. The most used epithelial cell adhesion molecule (EpCAM) was selected as the representative biomarker, and the anti-EpCAM antibody was grafted onto the conductive CNF-Chip via a voltage-sensitive gold-sulfur (Au-S) bond, which ensured the specific recognition and capture of EpCAM positive CTCs, and rapid release of CTCs through the cleavage of the Au-S bond under applying a low voltage (Figure 1d) [22,35]. The human colorectal cancer cell (HCT116) was used as the main target cell [36], and hepatocellular carcinoma cells (HepG2) and cervical cancer cells (HeLa) were also employed as controls to examine the capture efficiency of the constructed CNF-Chip-embedded microfluidic device [35]. Finally, the device was used to separate CTCs from clinical blood samples of different cancer patients.

## 2. Materials and Methods

### 2.1. Fabrication and Characterization of CNF-Chip

Firstly, the electrospinning technique was employed to fabricate polylactic acid (PLA) nanofibers. The PLA was added in a mixture solvent of N, N-Dimethylformamide (DMF) and dichloromethane (DCM; 2:1, *v*/*v*) to form the electrospinning solution with a PLA concentration of 12% (*w*/*v*). The dissolved PLA solution was poured into a 10 mL syringe, which was fixed on a microfluidic injection pump and connected to a 20-gauge steel needle. The electrospun PLA nanofibers were prepared under the conditions of an indoor temperature of 25 °C, an indoor humidity of 60%, a voltage of 15 kV, a 0.5 mL/h flow rate, and a collecting distance of 18 cm. The nanofibers were deposited onto the ITO substrate. Prior to collecting nanofibers, the ITO substrate was washed in advance by sonication in toluene (15 min), acetone (10 min), ethanol (15 min) and deionized water (20 min) in order, then treated with oxygen plasma for 10 min. The ITO with nanofibers was placed in a vacuum oven for standby. 

Then preliminarily modification of PLA nanofibers was applied. The sample was immersed in a 2 mg/mL dopamine solution (Tris-HCl buffer, 10 mM, pH ≈ 8.5) for 6 h to obtain the poly-dopamine (PDA) coating. After the reaction, the sample was washed with deionized water to remove excess PDA to obtain PLA/PDA. Then, the PLA/PDA was immersed in the HAuCl4 aqueous solution (0.5%, *w*/*v*), and 0.05 g/mL of KHCO_3_ and 5 mg/mL of glucose solution were added to grow AuNPs on PLA nanofibers in situ. After reaction for 6 h in a dark room, the PLA/PDA/AuNPs nanofibers were prepared and washed 3 times with deionized water and dried [37].

Next, a biofunctionalization procedure was implemented. The prepared PLA/PDA/AuNPs nanofibers were immersed in 2- mercaptoacetic acid (TGA) solution at a concentration of 1 mmol/mL for 24 h to introduce carboxyl groups in the surface of nanofibers via Au-S bonds, followed by washing with PBS. The carboxyl groups were then activated with 10 mmol/L EDC and 20 mmol/L NHS (MES buffer, 10 mM, pH = 5–6) for 4 h. Subsequently, the FITC-SA (2 μg/mL in PBS) was added on the surface of PLA/PDA/AuNPs/TGA nanofibers. After incubation at room temperature for 4 h, it was washed with PBS [38]. Finally, the 10 μg/mL biotin-labeled anti-EpCAM antibody solution was dropwise added onto the chip for functionalization for 1 h to obtain an antibody-modified nanofiber sample, namely the final CNF-Chip. The scanning electron microscope (JSM-7001F, JEOL, Ltd., Tokyo, Japan) was employed to observe the nanofibers in each step.

### 2.2. Preparation of Herringbone Microchannel and Assembly of the CNF-Chip-Embedded Microfluidic Device

The herringbone microchannel was designed by AutoCAD software. Then the herringbone groove patterns were fabricated onto a 10 μm-thick aluminum sheet via laser engraving by a laser marking machine (Han’s Laser UV-3s) and cut off, serving as the master template. The master template was fixed to a plane template by two-component epoxy adhesives, then evenly coated with a layer of trimethoxyheptadecylsilane for easy demolding, and dried in an oven at 70 °C for 2 h. Then 20 g PDMS (ratio of PDMS to curing agent in weight, 10:1) was poured onto the template, degassed via a pump, and baked in an oven for 2 h at 80 °C. After curing, carefully separate the cured PDMS from the template and punch the holes in the inlet and outlet locations to obtain a herringbone microchannel lid. Similarly, the PDMS was cast in a plane template with ITO to obtain the microchannel substrate with a chamber for fixing CNF-Chip. For characterization, a 15 nm layer of gold was sprayed onto the PDMS herringbone patterns in the microchannel lid. Both SEM and white light interferometer (Bruker Contour GT) were employed to observe the herringbone patterns. 

The herringbone microchannel lid and the microchannel substrate, between which the CNF-chip was embedded, were integrated immediately after plasma treatment, and a customized poly (methyl methacrylate) (PMMA) sealing set was used to seal the CNF-Chip-embedded microfluidic device.

### 2.3. Cell Capture Performance of CNF-Chip-Embedded Microfluidic Device

To evaluate the performance of the CNF-Chip-embedded microfluidic device for cell capture, 1 × 10^5^ HCT116 cells suspended in 1 mL PBS were pumped into the microfluidic device at a flow rate of 1 mL/h. After capture, CNF-Chip was flushed 3 times with PBS and taken out. The captured cells were fixed with 2.5% glutaraldehyde for 30 min and then stained with DAPI for 20 min. Finally, the captured cells on the CNF-Chip were observed under the fluorescence microscope and photographed in 5 random fields. The cell numbers in each image ($N_{n}$) were counted by ImageJ software to calculate the capture efficiency ($E_{C}$). The capture efficiency $E_{C}$ was calculated as follows [39]:

(1) $$N_{C}=\frac{N_{1}{+\ldots +N}_{n}}{n}\times \frac{S_{I}}{S}$$

(2) $$E_{C}\left(\%\right)=\frac{N_{C}}{N_{T}}\times 100\%$$

where $N_{C}$ is the number of captured cells, $S_{I}$ is the ITO area, S is the image area, and $N_{T}$ is the total number of cells pumped in, n = 5.

The effect of herringbone microchannel on capture performance was compared by setting up a static capture group and a flat-walled microchannel group. In the static capture group, the ITO substrate of CNF-Chip was replaced with a circular round cell slide (φ = 14 mm) and placed in a 24-well plate, and then 1 mL of HCT116 cell suspension (10^5^ cells/mL) was added to co-culture for 1 h. When studying the effect of flow rate on capture efficiency, only the flow rate was varied.

### 2.4. Cell Release Performance of CNF-Chip-Embedded Microfluidic Device

After cell capture, the washed CNF-Chip with cells was taken out and placed in an electrolytic cell with PBS as the electrolyte. The conductive CNF-Chip was taken as the working electrode, Ag/AgCl electrode was the reference electrode, and the platinum electrode was the counter electrode. A potential of −1.2 V was then applied by the electrochemical workstation (Zahner PP211) for 6 min. The cells were then released and collected by well plates. After that, the residual cells on the CNF-Chip were fixed with 2.5% glutaraldehyde for 30 min, stained with DAPI for 20 min, and observed under a fluorescence microscope. Five random fields of the sample were photographed. The obtained images were analyzed by ImageJ software, and the release efficiency ($E_{R}$) was calculated according to Equation (3):

(3) $$E_{R}\left(\%\right)=(1-\frac{N_{R}}{N_{T}{\times E}_{C}})\times 100\%$$

where ${\;N}_{R}$ is the number of residual cells on the CNF-Chip after release, $N_{T}$ is the total number of cells pumped in, and $E_{C}$ is the capture efficiency.

### 2.5. Isolation of CTCs from Cancer Patients’ Blood Samples

The study was performed in accordance with the tenets of the Declaration of Helsinki and approved by the ethics committee of the Third People’s Hospital of Chengdu and Southwest Jiaotong University. Blood samples from 11 cancer patients and 8 healthy volunteers were collected respectively and placed in ethylenediaminetetraacetic acid (EDTA) vacuum tubes. The blood samples were treated with ACK lysis buffer to remove erythrocytes and collect WBCs, including CTCs. Before cell capture, CNF-Chip was blocked with 2% BSA, which was beneficial for effectively reducing non-specific cell adhesion. The pretreated samples were then pumped into the CNF-Chip-embedded microfluidic device for CTC capture. After capture, the CNF-Chip with CTCs was washed with PBS 3 times and taken out. The CTCs on the chip were fixed with 2.5% glutaraldehyde at 4 °C for 2 h. 

Subsequently, the 3-color immunofluorescence staining was applied [18,40]. The 0.5% Triton X-100 was added and incubated with the cells at 4 °C for 10 min to increase cell membrane permeability. The cells were then incubated with 2% BSA at 4 °C for 30 min, washed with PBS 3 times and stained with 50 μL DAPI, 50 μL CD45 (20 μg/mL, green) and 50 μL CK (20 μg/mL, red) at 4 °C for 2 h. After washing, the CNF-Chip was observed under the fluorescence microscope to identify and count the CTCs (CTCs: DAPI+/CD45-/CK+; WBCs: DAPI+/CD45+/CK-).

## 3. Results and Discussion

### 3.1. Fabrication and Functionalization of CNF-Chip-Embedded Microfluidic Device

**Figure 2:**
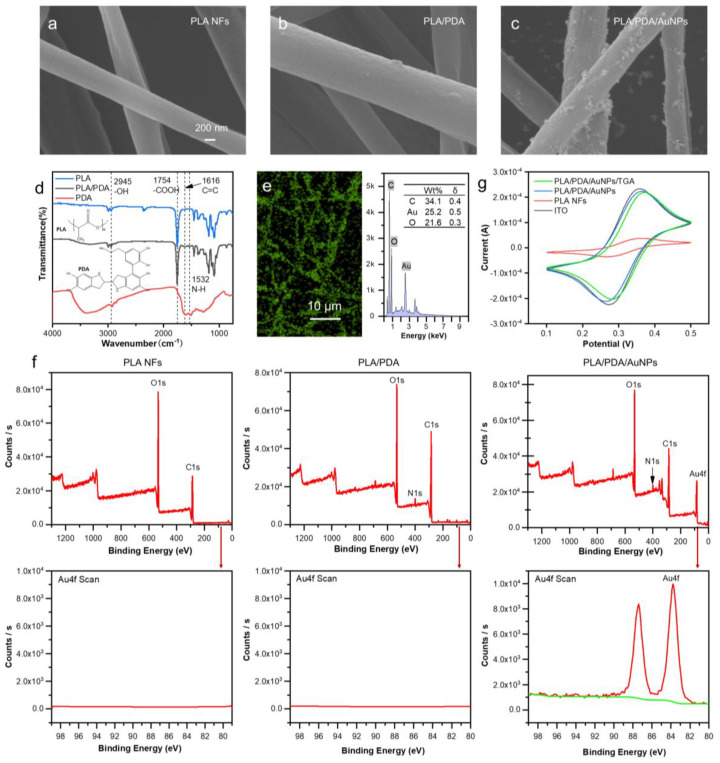
Preparation and characterization of the CNF-Chip. (**a**–**c**) SEM images of PLA NFs, PLA/PDA, and PLA/PDA/AuNPs on the substrates. (**d**) FTIR spectra of PLA, PLA/PDA and PDA. (**e**) EDS elemental mapping for Au on the surface of PLA/PDA/AuNPs nanofibers and corresponding content. (**f**) Elemental analysis of PLA, PLA/PDA, PLA/PDA/AuNPs fiber membrane surfaces by X-ray photoelectron spectroscopy. (**g**) Cyclic voltammograms of ITO substrate, PLA NFs, PLA/PDA/AuNPs and PLA/PDA/AuNPs/TGA in K_3_[Fe(CN)_6_] electrolyte with a scan rate of 10 mV/s.

The CNF-Chip was prepared by depositing electrospun PLA nanofibers (NFs) onto an ITO substrate, followed by the PDA modification, the in situ growth of AuNPs and the bio-functionalization procedure. To obtain a dense nanofibrous substrate, the deposition time was optimized as 2 h, and the porosity reached 34.7% (Figure S1). When applied longer deposition time over 2 h, the deposited PLA NFs would easily detach from the ITO substrate when immersed in an aqueous solution. Then the preliminary modification procedures were applied (Figure 1a,b). As shown in Figure 2a–c, The SEM images of the fibers revealed that the surface of PLA NFs changed from smooth to rough after PDA modification, and a large number of nanoparticles appeared on the surface after in situ growth of AuNPs. Accordingly, the color of the fibers changed from white to dark brown to gray-purple (Figure S2a), and the diameter of the fibers changed from 359.9 ± 2.9 nm (PLA NFs) to 374.7 ± 5.9 nm (PLA/PDA) to 390.9 ± 7.1 nm (PLA/PDA/AuNPs; Figure S2b).

Additionally, as shown in Figure 2d, after modification with PDA, catechol and amine functional groups were introduced onto PLA NFs, which was conducive to the subsequent modification [41]. As shown in Figure 2e, the fiber surface was covered with gold elements after growing AuNPs in situ, demonstrating the successful gold coating and providing sufficient reaction sites for the grafting antibodies by Au-S bonding. According to further XPS analysis of the elements on the fiber surface (Figure 2f), both the PLA/PDA and PLA/PDA/AuNPs nanofibers displayed N1s peaks, and the PLA/PDA/AuNPs nanofiber displayed Au binding energy at 84 eV. The results mentioned above demonstrated that the PLA/PDA/AuNPs nanofiber surface was successfully coated with gold. The conductivities of fibers at different stages were further characterized by cyclic voltammetry. The ITO substrates covered with different nanofibers were attached to the working electrodes and immersed in K_3_[Fe(CN)_6_] electrolyte for voltammetric testing. The results (Figure 2g) showed that the conductivity of the ITO substrate reduced after the deposition of PLA NFs, which was nearly restored after the modification with PDA and AuNPs. These results suggested that the introduction of Au-endowed PLA NFs with good conductivity. In addition, after further TGA modification, the cyclic voltammograms (CV) curves only showed a slight decrease in conductivity. To eliminate the interference of the ITO glass itself and further confirm the TGA modification, the CV curves of fibers on ordinary glass substrate were also tested, as shown in Figure S3. The CV curve displayed that the current was decreased after the introduction of TGA because the TGA may hinder electron transfer [32]. These results indicated that the TGA was successfully modified onto the surface of PLA/PDA/AuNP nanofibers. 

**Figure 3:**
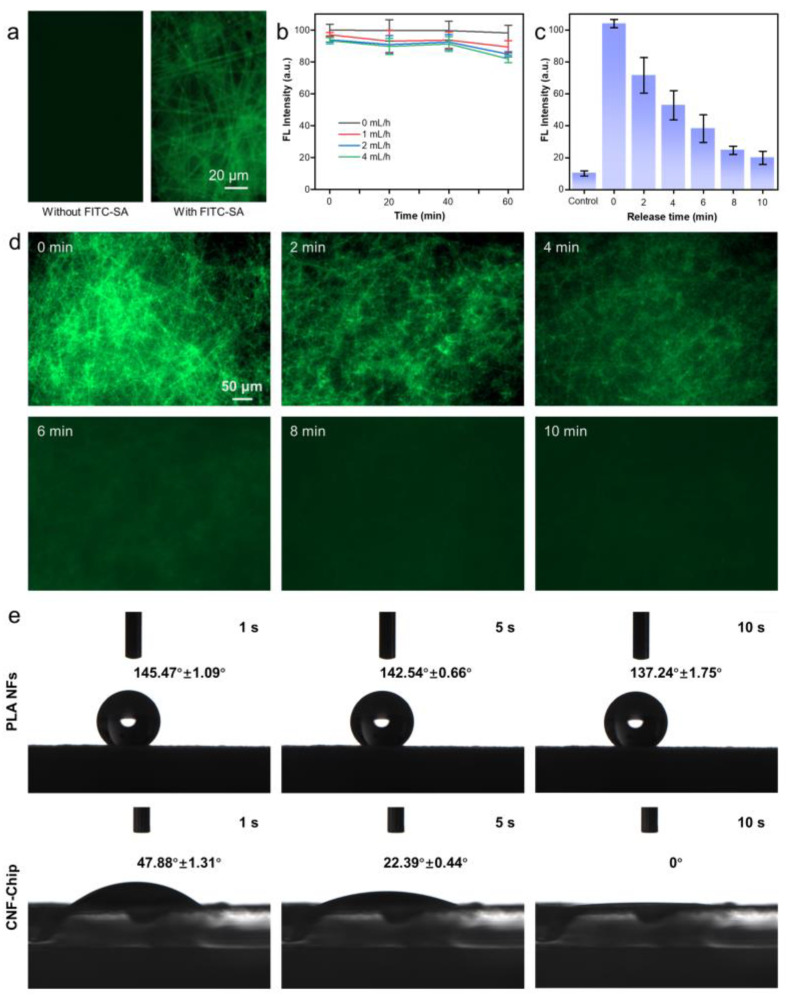
Biofunctionalization and characterization of the CNF-Chip. (**a**) Fluorescent images of PLA/PDA/AuNPs without and with FITC-SA. (**b**) Fluorescence intensity of the AuNPs-coated nanofibers with FITC-SA under different flow rates. (**c**) Fluorescence intensity of the AuNPs-coated nanofibers with FITC-SA after applying voltage for different times. (**d**) Fluorescent images of FITC-SA modified nanofibers after electrochemical release for 2, 4, 6, 8, and 10 min, respectively. (**e**) The water contact angle of PLA NFs and CNF-Chip.

The introduction of TGA via Au-S bond brought the carboxyl groups, which can form amide bonds with amino groups in streptavidin (SA; Figure S4). The SA was used to specifically bind the biotin-labeled anti-EpCAM molecules. In this study, the fluorescein isothiocyanate-labeled SA (FITC-SA) was employed to visualize the distribution of the reaction sites, as shown in Figure 3a. It could be seen that after FITC-SA modification, all fibers showed strong fluorescence. Furtherly, to detect the stability of fibers on the ITO substrate, as shown in Figure 3b, ITO substrate integrated with FITC-SA-labeled PLA/PDA/AuNPs fibers were placed in the microfluidic channel and rinsed with PBS at flow rates of 0, 1, 2, and 4 mL/h. After continuous rinsing for 60 min, only a slight decrease occurred in the fluorescence intensity of fibers, which could be attributed to the elution of a small portion of unbounded FITC-SA and the fading of fluorescence with time. The results showed that through preliminary modification, the reaction sites on the fiber surface for grafting biotin-labeled anti-EpCAM molecules were abundant and distributed evenly throughout the surfaces of fibers. Here, although the EpCAM was employed as a model biomarker for CTC targeting, and the anti-EpCAM antibodies were coated onto the rough nanointerface of the device, the biomarkers can be instead with other types of antibodies, like antibodies for HER2, CD26 or CD44, etc. [13], which requires further research in the future.

Subsequently, the electrochemical release ability of the preliminarily biofunctionalized CNF-Chip was further characterized. It was well known that the Au-S bond was sensitive and breakable at the negative potential [32]. Thus, a voltage of −1.2 V was applied to PLA/PDA/AuNPs labeled with FITC-SA through an electrolytic cell for 0, 2, 4, 6, 8 and 10 min (Figure 3c,d,). PLA/PDA/AuNPs without FITC-SA were used as the control. It could be found that when electrochemical stimulation was shorter (within 4 min), clear fiber structures with green fluorescence could be observed in Figure 3d. While the fluorescence intensity decreased dramatically by 70.2% when 6 min of electrochemical stimulation was applied, and the fiber structures became blurred. After 6 min, no fiber structures could be found in the fluorescence images, and the corresponding fluorescence intensity seemed to decrease only a little. Therefore, applying voltage for 6 min was selected in the subsequent experiments. In addition, the hydrophobic PLA fibers became hydrophilic after a series of modifications by PDA, AuNPs and biofunctionalization (Figure 3e). Besides, the cell viability was still as high as 95% after 24 h’s incubation with PLA NFs or CNF-Chip, indicating that the employed materials had excellent biocompatibility and the modification processes would not bring any bad effects (Figure S5).

**Figure 4:**
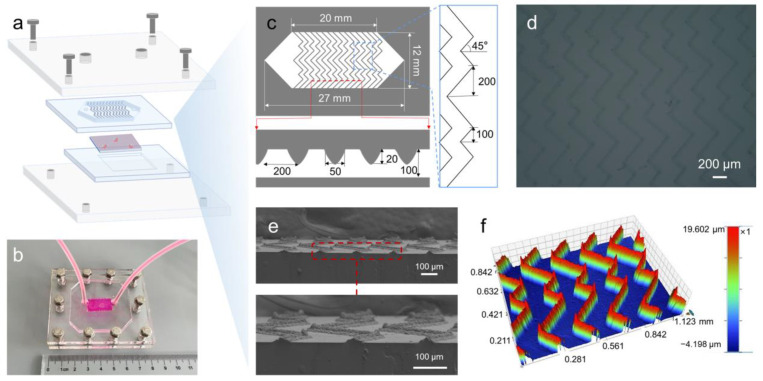
Preparation and characterization of the microchannel. (**a**) Schematic illustration of CNF-Chip-embedded microfluidic device. (**b**) Photograph of the CNF-Chip-embedded microfluidic device. (**c**) Schematic illustration for parameters of the herringbone microchannels. (**d**) Photograph of PDMS microchannel of the device. (**e**) SEM images of the herringbone micropattern cross-section. (**f**) The 3D imaging of the herringbone microchannel lid shows the height at 20 μm.

According to a previous report [42], the microfluidic channel with a herringbone pattern can promote the passive mixing of target cells by generating microvortexes, disrupting streamlines, and maximizing the probability of collision between the cells and the antibody-coated substrate, thereby increasing the capture efficiency. Figure 4a,b displayed the schematic illustration and photograph of the CNF-Chip-embedded microfluidic device, which was composed of the CNF-Chip, the PDMS microchannel with herringbones and a sealing set. An aluminum sheet with negative herringbone patterns, printed by a laser marking machine, was used as the master template, as shown in Figure S6. The designed dimensions of the PDMS herringbone microchannel are shown in Figure 4c. The herringbones were on the upper surface of the microchannel. The overall height of the microchannel (h) was 100 μm. For the herringbone pattern, the groove height was 20 μm, the angle between the herringbones and the axis of the channel (θ) was 45°, and the spacing between each groove was 200 μm. The inlet and outlet were located at the ends of the microchannel, respectively. The microchannel was made by a cast molding process. The morphologies of the final herringbone patterns on the upper surface of the PDMS microchannel are shown in Figure 4d. Figure 4e,f further characterized the height and physical appearance of herringbone patterns. According to previous reports [8,9,43,44,45], to prepare devices based on cavitation inception pressures or other biophysical properties of CTCs, complicated lithography processes were often implemented to form complex geometry. Meanwhile, the preparation of the CNF-Chip-embedded microfluidic device was easier, for which only a laser marking machine and the electrospinning equipment with simple fabrication procedures were needed. In addition, no other extra device was needed during usage.

### 3.2. Performance of CNF-Chip-Embedded Microfluidic Device for CTC Capture

**Figure 5:**
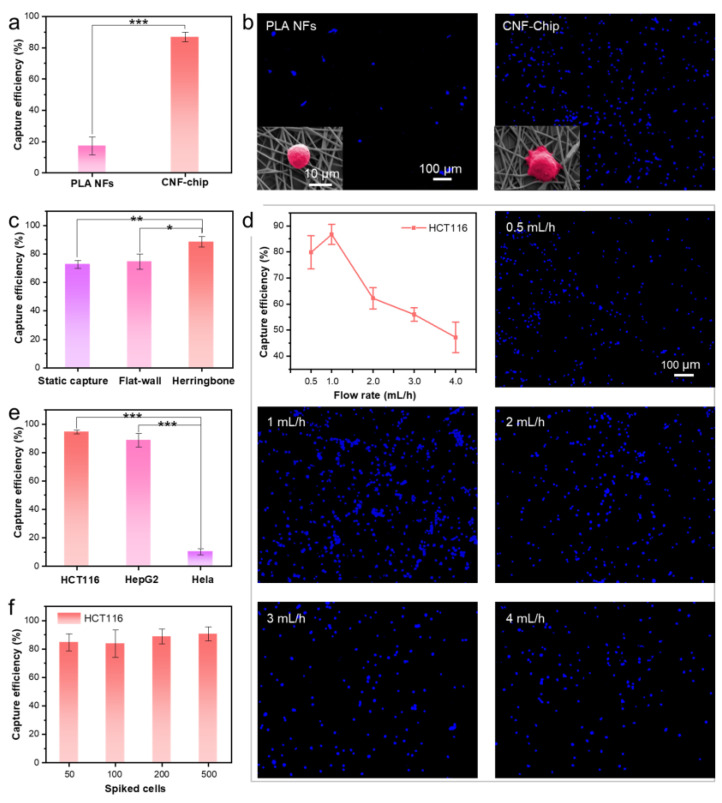
CTC Capture efficiency of CNF-Chip-embedded microfluidic device. (**a**) CTC Capture efficiency of the PLA NFs and the CNF-Chip-embedded microfluidic device (1 × 10^5^ cells/mL, 1 mL/h). (**b**) Fluorescence images with inserted SEM images. (**c**) Capture efficiency in static conditions and the CNF-Chip-embedded microfluidic device with or without herringbone pattern (1 × 10^5^ cells/mL, 1 mL/h). (**d**) Capture efficiency and representative fluorescence images of captured CTCs under different rates. (**e**) Capture efficiency of CNF-Chip-embedded microfluidic device for HCT116 (EpCAM++), HepG2 (EpCAM+) and HeLa (EpCAM−; 1 × 10^5^ cells/mL, 1 mL/h). (**f**) Capture efficiency of CNF-Chip-embedded microfluidic device for HCT116 cells spiked in PBS with different concentrations under 1 mL/h.

The CTC capture performance of the CNF-Chip-embedded microfluidic device was investigated by using artificial cell suspensions (capture efficiencies are shown in Table S1). Several factors that may affect the capture efficiency of the device were considered, including the biofunctionalization of the fibers, the herringbone microchannel, flow rate, cell type and cell concentration. Firstly, to investigate the effect of biofunctionalization of the fibers, CNF-Chip and unmodified PLA NFs were both integrated into the microfluidic device separately for CTC capture. As compared with the PLA NFs group, the capture efficiency of the CNF-Chip group for HCT116 cells was significantly increased from 17.2% to 86.9% (Figure 5a). As shown in Figure 5b, the fluorescence images showed that the CNF-Chip captured obviously more cells. Furtherly, it could be seen from the SEM images in the inserts that the captured cells in the CNF-Chip extended more pseudopodia, presenting enhanced adhesion to the fibers. These results suggested that the CNF-Chip greatly improved the capture efficiency, which may be attributed to the fact that both of the rough nanostructures provided by the PDA/AuNPs modified nanofibers and the strong affinity of antibodies on the surface of CNF-Chip, could promote the interaction between targeted cells and the nanofibrous substrate, eventually improving the capture efficiency.

Next, to investigate the effect of herringbone microchannel on the capture performance, a static capture group and a flat-walled microchannel group were set for comparison. As shown in Figure 5c and Figure S7, the capture efficiency of the static group was only 72.7%, and the capture efficiency of the flat-walled microchannel was 74.7%. While the capture efficiency of the CNF-Chip increased to 88.6%, indicating that the introduction of a herringbone microfluidic chaotic mixer increased the number of cell-surface interactions in the device. The reason may be that the laminar streamlines that cells traveled in through the flat-walled microchannel were disrupted and changed to a chaotic mode in the herringbone microchannel, increasing the collision probability between the cells and the nanofibrous substrate [46].

The effect of flow rate on the capture performance was also studied. As shown in Figure 5d, the capture efficiency was 79.9% at a flow rate of 0.5 mL/h, 86.7% at 1 mL/h, 62.2% at 2 mL/h, 56.0% at 3 mL/h and 47.2% at 4 mL/h, respectively, suggesting that the overall capture efficiency decreased with increasing flow rate and reached the maximum value at 1 mL/h. Therefore, the optimal flow rate for the CNF-Chip-embedded microfluidic device in the subsequent experiment was chosen as 1 mL/h.

Furthermore, since the expression level of EpCAM varied in different cells [46], the capture performance of CNF-Chip-embedded microfluidic device on different types of cancer cells was evaluated (Figure 5e). The EpCAM positive cells, HCT116 cells (high expression, EpCAM++) and HepG2 cells (low expression, EpCAM+), and the EpCAM negative (EpCAM−) HeLa cells were all included [35,36]. The results showed that under the flow rate of 1mL/h, the capture efficiency for HCT116 (EpCAM++) was as high as 94.6% and that for HepG2 (EpCAM+) was 88.6%. At the same time, the capture efficiency for HeLa (EpCAM−) was only 10.3%. These results demonstrated that the CNF-Chip-embedded microfluidic device possessed specific and excellent capture ability to the EpCAM-positive cells.

Finally, the concentration of the cell suspension was reduced to test the device in a situation more similar to the actual clinical sample. As shown in Figure 5f, even if the concentration changes from 50 to 500 cells/mL, the capture efficiency could still remain over 80%. Actually, the CNF-Chip-embedded microfluidic device can detect the presence of CTC down to 50 CTCs/mL and maintained a capture efficiency of 84.7%, indicating that the CNF-Chip-embedded microfluidic device was still sensitive to the targeted cells at low concentration.

### 3.3. Performance of CNF-Chip-Embedded Microfluidic Device for CTC Release

**Figure 6:**
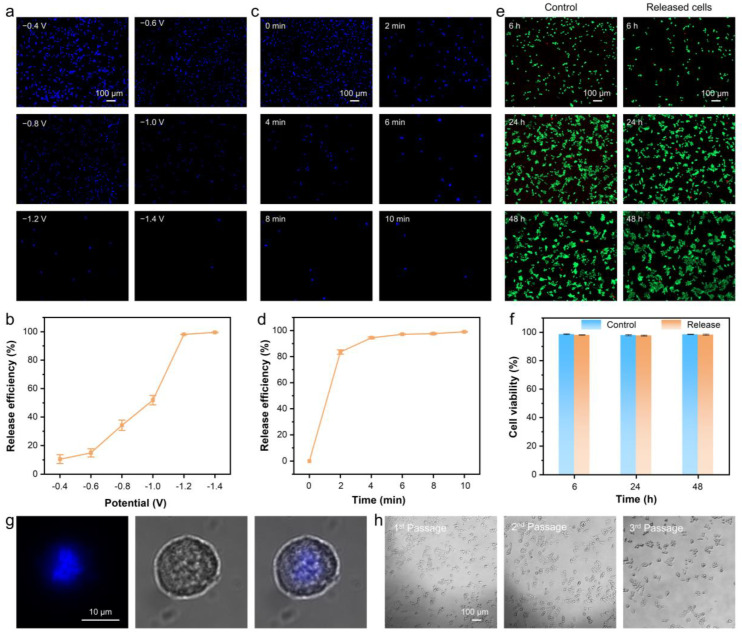
The electrochemical release of captured HCT116 cells. (**a**) Fluorescence images and (**b**) release efficiency of captured HCT116 cells on CNF-Chip after applying different negative voltages for 6 min. (**c**) Fluorescence images and (**d**) release efficiency of CTCs on CNF-Chip after applying a potential of −1.2 V for different times. (**e**) Fluorescence images of control cells and released cells. (**f**) Corresponding cell viability in (**e**). (**g**) Fluorescence images of released cells stained with DAPI. (**h**) Microscopy images of released cells cultured for 1 to 3 passages.

Since the electrically sensitive Au-S bond was employed to bind the anti-EpCAM antibody on the conductive fibers, the release of captured cells should be achieved by simply applying a certain voltage (release efficiencies are shown in Table S2). According to a previous report [33], CNF-Chips were applied with different negative voltages (Figure 6a,b), and the release efficiency increased with an increase in the absolute value of negative voltage. When the voltage reached −1.2 V, the release efficiency reached 98.1% within only 6 min. In addition, when fixed the voltage at −1.2 V, the release efficiency increased with time (Figure 6c,d). When the applying voltage for 2 min, the release efficiency already reached over 80%, and after 6 min (97.1%), the release efficiency increased slowly. Considering that lower applied voltage and shorter electrochemical stimulation time should be more beneficial to cell viability [47], the release conditions were set as −1.2 V and 6 min in future experiments.

In fact, the capture and release process may take about 70 min in total, suggesting that the on-chip residence times of cells were less than 70 min, which matched the limited half-life of CTCs, namely between 1 and 2.4 h, according to the previous report [48]. Then the viability of the released cells was further determined, as shown in Figure 6e,f. The released cells were collected and cultured for a period of time, then evaluated by live/dead staining assay. It could be seen that, as compared to the control group (cells artificially seeded onto the cell culture plate without any treatment), only a few dead cells (red ones) appeared during the incubation of 48 h. The corresponding quantitative data showed that the survival rate of the released cells after 48 h’s culture was still above 97%, which was equivalent to that of the control group, indicating that the cells still had good viability after capture and release procedures (Figure 6f). What’s more, the structure of released cells was intact, and there was no significant morphological change (Figure 6g). After passaging the released cells three times, the cells remained alive and had a healthy appearance (Figure 6h). These results suggested that the rapid and on-demand release of targeted cells could be achieved by electrochemical stimulation, which brought less damage to captured cells and maintained higher cell viability of released CTCs, benefiting the in vitro CTC amplification and downstream analysis. While the captured CTCs by the common biophysical properties-based approaches are often no longer intact after being subjected to shear forces, making subsequent CTCs identification and detection difficult [5].

### 3.4. Capture of CTCs from Clinical Blood Samples of Patients

**Figure 7:**
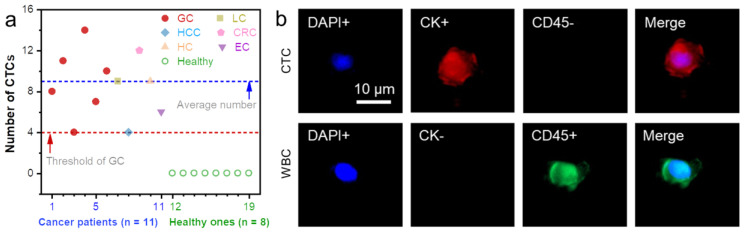
CTCs detection for clinical blood samples from different cancer patients. (**a**) Quantitative data of CTCs from blood samples of cancer patients and healthy volunteers. (Gastric cancer (GC), lung cancer (LC), hepatocellular carcinoma (HCC), colorectal cancer (CRC), hilar cholangiocarcinoma (HC), endometrial carcinoma (EC)). (**b**) The representative circulating tumor cell (DAPI+/CK+/CD45-) and WBC (DAPI+/CK-/CD45+) captured by the CNF-Chip-embedded microfluidic device.

To test the performance of the CNF-Chip-embedded microfluidic device on clinical samples, blood samples from eight healthy volunteers and 11 patients with different cancers at various stages were collected and used. The basic clinicopathological data are shown in Table S3. Considering the interference of the large number of WBCs in the blood sample, CNF-Chip was blocked with BSA before CTC capture and tested by the blood sample from one healthy volunteer, as shown in Figure S8. The results showed that after BSA blocking, the number of WBCs significantly decreased. For blood samples from patients, the captured cells on the CNF-Chip were subjected to three-color immunofluorescence staining. The DAPI+/CK+/CD45- cells were defined as tumor cells, and DAPI+/CK-/CD45+ cells were defined as WBCs [49]. Based on this standard, the CTCs isolated respectively from those 19 blood samples were stained and analyzed, as shown in Figure 7a and Table S3. A representative fluorescent image of captured cells from patient No. 9 was shown in Figure S9, indicating that the rare CTC could still be captured from the blood. The labeled CTC and one randomly picked WBC were zoomed in, as shown in Figure 7b. It suggested that no CTCs were found in the blood samples from eight healthy volunteers, while different amounts of CTCs were detected in blood samples from six gastric cancer (GC) patients, a lung cancer (LC) patient, a hepatocellular carcinoma (HCC) patient, a colorectal cancer (CRC) patient, a hilar cholangiocarcinoma (HC) patient, and an endometrial carcinoma (EC) patient, respectively. The threshold number of CTCs was significantly correlated with clinical stage III-IV gastric cancer [50]. Our results showed that the mean number of CTCs in patients with gastric cancer was nine, and all patients with stage III-IV gastric cancer had a number of CTCs ≥ 4 cells/mL. In addition, the overall average CTC number of all the cancer patients was around nine. Though more clinical samples should be detected to confirm the sensitivity, those results still suggested that the CNF-Chip-embedded microfluidic device had excellent potential for clinical applications in CTCs isolation and analysis.

## 4. Conclusions

In conclusion, though our device could not capture all CTCs, it offered a new strategy to simplify the combination of micro-geometries and nanostructures and the surface modification for developing microfluidic devices for effective capture and rapid release of CTCs. The integration of the herringbone microchannel and the anti-EpCAM-coated conductive nanofiber chip greatly enhanced the topological interaction between the targeted cells and the interface of the substrate, increasing the capture efficiency for EpCAM-positive CTCs. At the same time, the use of the Au-S bond for anti-EpCAM antibody grafting ensures the rapid and on-demand release of CTCs under low voltage. This microfluidic device was successfully applied to isolate the CTCs in blood samples from different cancer patients, indicating its great potential in the diagnosis, treatment monitoring and prognosis of cancer patients. In the future development, this work may be associated with the Internet of Things to realize integration and automation of CTC detection [51].

## Data Availability

Not applicable.

## Supplementary Materials

The following supporting information can be downloaded at: https://www.mdpi.com/article/10.3390/bios13050497/s1 [52,53]. Figure S1. (a) SEM images and (b) corresponding fiber porosities of electrospun PLA NFs under different deposition times. Figure S2. (a) Images of conductive ITO substrate and different fibers (PLA NFs, PLA/PDA, PLA/PDA/AuNPs) on the substrates. (b) Fiber diameter statistics for dif-ferent fibers. Figure S3. CVs recorded for K_3_[Fe(CN)_6_] electrolyte at AuNPs-coated nanofibers on glass substrate with or without TGA modification. Figure S4. Activation and amination reaction processes. Figure S5. (a) Fluorescence images of cells without any treatment (control), and cells incubated with PLA NFs or CNF-chip for 24 h. All cells were stained with PI/CAL-AM. (b) The corresponding cell viabilities after 24 h in different groups. Figure S6. (a) The 2D and (b) 3D imaging of the master template with white light in-terferometer showing the depth of the grooves at 20 μm. Figure S7. Fluorescence images of CTCs captured in static conditions and the CNF-Chip microfluidic device with flat-wall or herringbone pattern. Figure S8. Representative fluorescence images of WBCs from blood sample of one healthy volunteer, captured by the CNF-Chip-embedded microfluidic device without or with BSA modification. Figure S9. Representative images of CTCs from a colorectal cancer patient’s blood sample stained with antibodies against CK (red) and CD45 (green), and DAPI (blue). Table S1. Capture efficiency of CNF-Chip-embedded microfluidic device. Table S2. Release efficiency of CNF-Chip-embedded microfluidic device. Table S3. Quantitative analysis of Cancer patients’ blood samples.
