# Supplementary material for: Conductive Nanofibers-Enhanced Microfluidic Device for the Efficient Capture and Electrical Stimulation-Triggered Rapid Release of Circulating Tumor Cells
Source: Biosensors (Basel). 2023 Apr 25;13(5):497. doi: 10.3390/bios13050497 (PMC10216647; doi:10.3390/bios13050497)
Supplement: Supplementary file 1 [file biosensors-13-00497-s001.zip › biosensors-2302599-supplementary.pdf]

Supplementary Materials

Conductive Nanofibers-Enhanced Microfluidic Device for Efficient Capture and Electrical Stimulation-Triggered Rapid Release of Circulating Tumor Cells

Supplementary Experiments:

Materials：

Polylactic acid (60-70 KDa) was synthesized by our own laboratory. N,N-Dimethylformamide (DMF), dichloromethane (DCM), anhydrous ethanol (99.8%), hydrochloric acid (AR), potassium bicarbonate ($KHCO_3$) and glucose were purchased from Chengdu Kelong Chemical Reagent Co., Ltd. Polydimethylsiloxane (PDMS) (Sylgard 184) was purchased from Dow Corning Co., Ltd. Trimethoxyheptafluorodecylsilane (98%), dopamine hydrochloride (98%), trimethylolaminomethane (98%), chloroauric acid ($HAuCl_4$), 2-mercaptoacetic acid (TGA), and MES buffer were purchased from Aladdin Co., Ltd. The 1-ethyl-3-(30-dimethylaminopropyl) carbodiimide (EDC), N- hydroxysuccinimide (NHS) and glutaraldehyde (GA) were purchased from Adamas Co., Ltd. Fluorescein isothiocyanate (FITC)-labeled streptavidin (FITC-SA) was purchased from Tianjin Silang Technology Development Co., Ltd. Biotin-labeled anti-EpCAM antibody and FITC-conjugated anti-CD45 (CD45) were purchased from Abcam. Immunostaining with phycoerythrin (PE)-conjugated anti-pan cytokeratin (CK) was purchased from Cell Signaling Technology. The 4,6-diamidino-2-phenylindole (DAPI), propidium iodide (PI) and calcein-AM (CAL-AM) were purchased from Sigma-Aldrich Co., Ltd (USA). Two-component epoxy adhesives (5 Minute Epoxy) were purchased from Devcon Co., Ltd. Bovine serum albumin (BSA) was purchased from Ruji Biotechnology Co. ACK Lysis Buffer was purchased from Solarbio Life Sciences Co. Ltd (Beijing, China). Ltd (Shanghai, China). Indium–tin oxide (ITO) conductive film glass (7-10 Ω, 20×12×1.1 mm) was purchased from Guluo Glass Co., Ltd (Luoyang, China). A polymethyl methacrylate (PMMA) sealing device (80×70×4.5 mm) was purchased from Chixin Biotechnology Co., Ltd (Anhui, China).

Stability of CNF-Chip

To visualize and characterize the reaction sites on the fibers, before biotin-labeled anti-EpCAM was modified, FITC-SA was employed to bind on fibers. The PLA/PDA/AuNPs fibers bound with FITC-SA were integrated in the microfluidic device and then rinsed by PBS solutions with flow rates of 0 mL/h, 1 mL/h, 2 mL/h and 4 mL/h, and the fluorescence intensities were observed and recorded under a fluorescence microscope (Axio Observer, Zeiss, Germany) every 20 minutes to study the stability of FITC-SA.

Breakage of Au−S bond

The study of the release performance of the CNF-Chip was achieved by applying different voltages. The PLA/PDA/AuNPs fibers bound with FITC-SA were applied with a voltage of -1.2 V for 0 min, 2 min, 4 min, 6 min, 8 min and 10 min, respectively. Then, they were washed with PBS, and the fluorescence intensities of the fibers were observed and recorded after the corresponding times.

Cell culture

Three types of human tumor cells were selected, including colorectal cancer cell HCT116 (EpCAM++), hepatocellular carcinoma cells HepG2 (EpCAM+) and cervical cancer cell Hela (EpCAM−). The cells were cultured in Dulbecco's modified Eagle medium

(DMEM) with the addition of 10% fetal bovine serum and 1% penicillin–streptomycin solution for one to two days in 37 ℃ cell incubator containing 5% CO_2 [1].

Cytocompatibility of CNF-Chip

The cytocompatibility of the CNF-Chip was evaluated using the live/dead staining assay [2]. Cell suspension with a concentration of 5×10^4 cells /mL was added into the 24-well plate. After the cells adhered to the wall, unmodified PLA NFs and modified CNF-Chip samples were added to co-incubate with the cells, and the samples were cut into small blocks of 1 cm × 1 cm. At the same time, the group of cells without any treatment was considered as control. After 24 h, the added materials and culture medium was removed. Then, the cells were stained with CAL-AM/PI for 20 min, observed and counted for the number of live (green) dead (red) cells by fluorescence microscopy and calculated the cell viability.

Viability of the released CTCs

The released cells in PBS were centrifuged and re-suspended, then seeded in well plates and incubated for 48 h. A control group was set to characterize the normal survival rate of cells without capture and release process. After a certain culture time, live/dead staining was applied and cells were observed by fluorescence microscopy. Similar image analysis was carried out via ImageJ software as described in the *Cytocompatibility of CNF-Chip* section.

As the cells grew up to 80%, they were passaged and inoculated into another new well plate for the culture. The cells that had been continuously passaged for 3 generations, and cells from each generation, were observed and photographed under the bright field of the microscope.

Supplementary Figures:

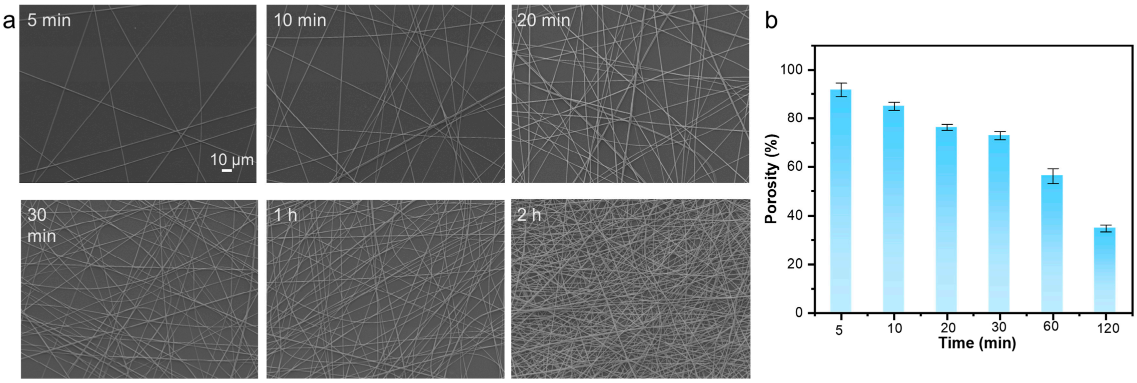

Figure S1. (a) SEM images and (b) corresponding fiber porosities of electrospun PLA NFs under different deposition times.

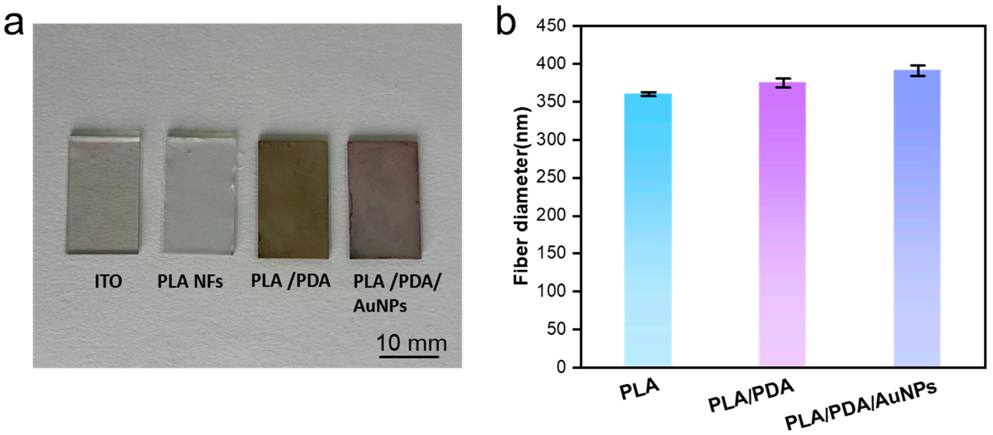

Figure S2. (a) Images of conductive ITO substrate and different fibers (PLA NFs, PLA/PDA, PLA/PDA/AuNPs) on the substrates. (b) Fiber diameter statistics for different fibers.

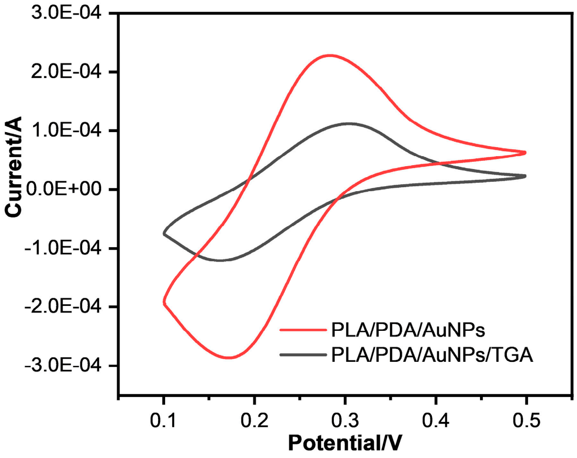

Figure S3. CVs recorded for $K_3[Fe(CN)_6]$ electrolyte at AuNPs-coated nanofibers on glass substrate with or without TGA modification.

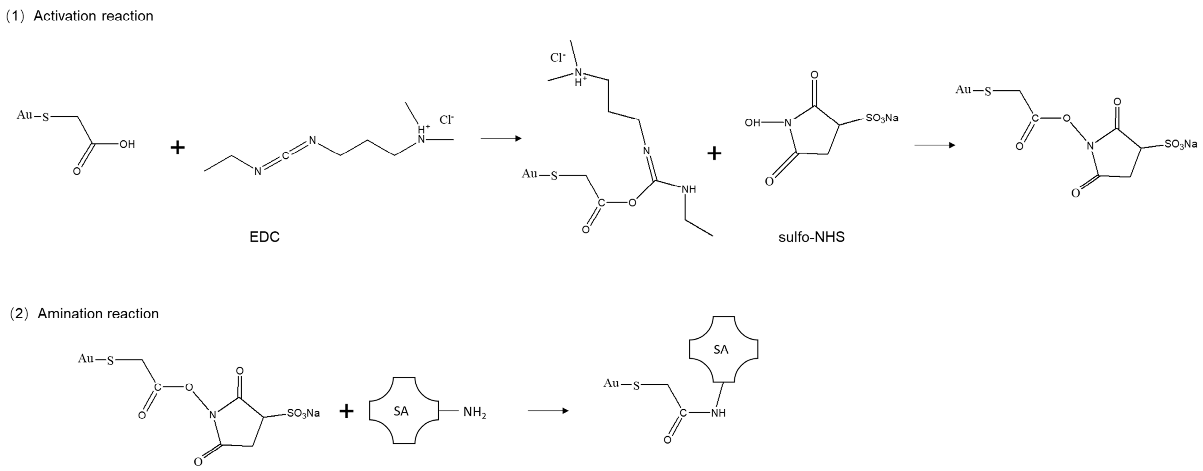

Figure S4. Activation and amination reaction processes.

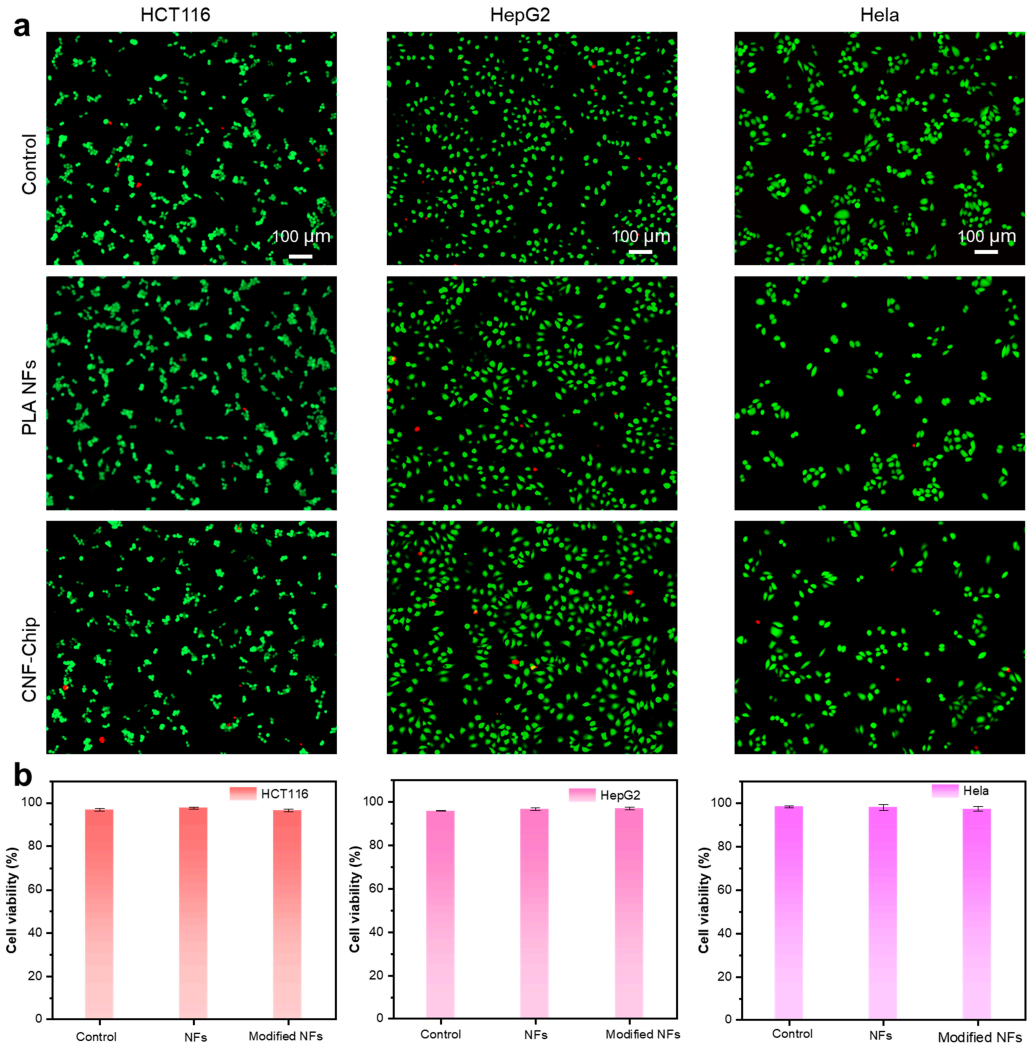

Figure S5. (a) Fluorescence images of cells without any treatment (control), and cells incubated with PLA NFs or CNF-chip for 24 h. All cells were stained with PI/CAL-AM. (b) The corresponding cell viabilities after 24 h in different groups.

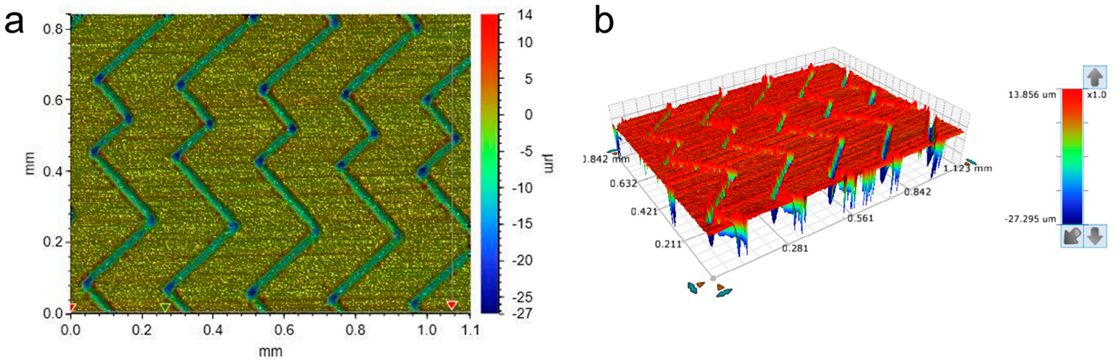

Figure S6. (a) The 2D and (b) 3D imaging of the master template with white light interferometer showing the depth of the grooves at 20 μm.

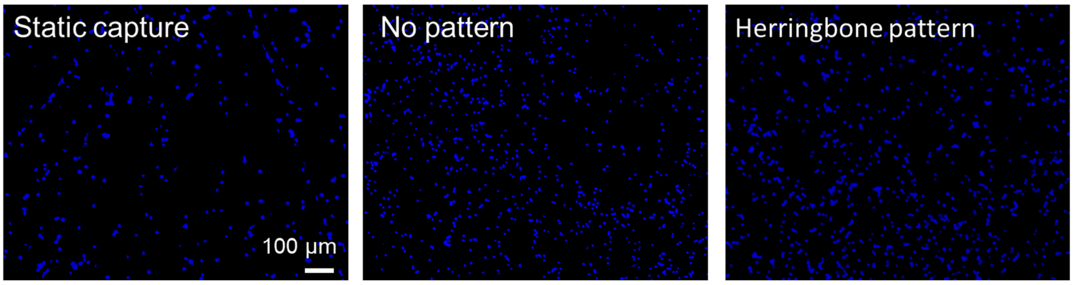

Figure S7. Fluorescence images of CTCs captured in static conditions and the CNF-Chip microfluidic device with flat-wall or herringbone pattern.

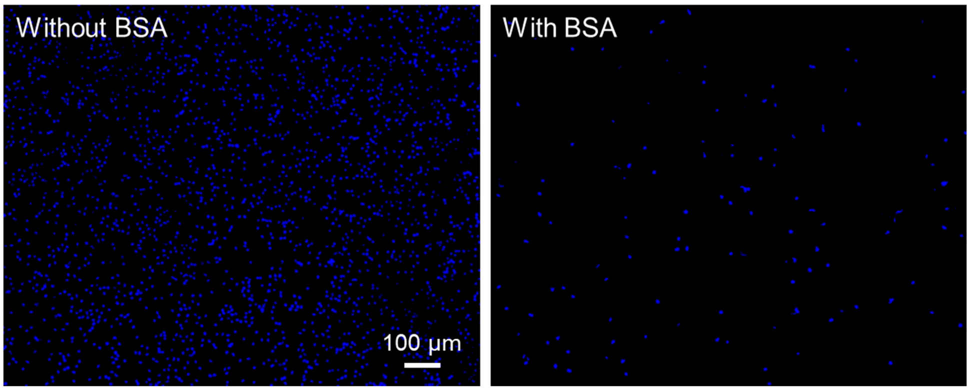

Figure S8. Representative fluorescence images of WBCs from blood sample of one healthy volunteer, captured by the CNF-Chip-embedded microfluidic device without or with BSA modification.

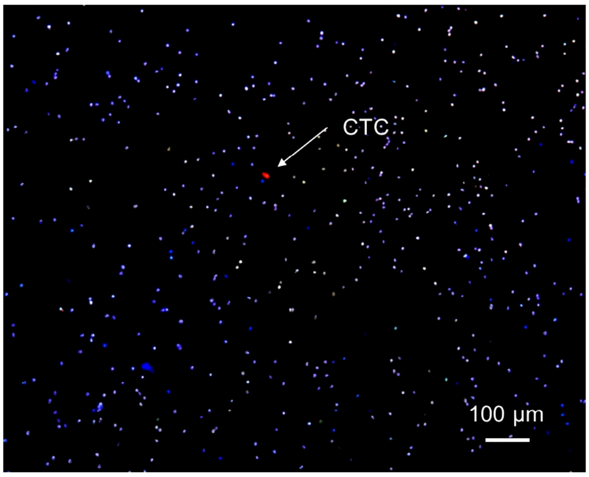

Figure S9. Representative images of CTCs from a colorectal cancer patient's blood sample stained with antibodies against CK (red) and CD45 (green), and DAPI (blue).

Supplementary Tables:

Table S1. Capture efficiency of CNF-Chip-embedded microfluidic device.

Device	Cell type	Concentraion (cells/mL)	Flow rate (mL/h)	Capture efficiency (%)
PLA NFS	HCT116	1×10^5	1	17.2±5.8
CNF-Chip	HCT116	1×10^5	1	86.9±3.0
Static capture	HCT116	1×10^5	1	72.7±2.9
Flat-wall	HCT116	1×10^5	1	74.7±5.3
Herringbone	HCT116	1×10^5	1	88.6±3.7
CNF-Chip-embedded microfluidic device	HCT116	1×10^5	0.5	79.9±6.4
	HCT116	1×10^5	1	86.7±3.9
	HCT116	1×10^5	2	62.2±4.1
	HCT116	1×10^5	3	56.0±4.1
	HCT116	1×10^5	4	47.2±5.9
	HCT116	1×10^5	1	94.7±1.4
	HepG2	1×10^5	1	88.6±4.9
	HeLa	1×10^5	1	10.3±2.3
	HCT116	50	1	84.7±6.1
	HCT116	100	1	83.7±9.6
	HCT116	200	1	88.8±5.1
	HCT116	500	1	90.7±5.1

Table S2. Release efficiency of CNF-Chip-embedded microfluidic device.

Voltage (V)	Release time (min)	Release efficiency (%)
-0.4	6	10.6±3.1
-0.6	6	14.9±2.8
-0.8	6	34.2±3.6
-1.0	6	51.9±3.3
-1.2	6	98.1±0.1
-1.4	6	99.5±0.2
-1.2	0	0
-1.2	2	83.5±1.7
-1.2	4	94.4±0.7
-1.2	6	97.1±0.3
-1.2	8	97.6±0.7
-1.2	10	99.0±0.3

Table S3. Quantitative analysis of Cancer patients' blood samples.

**Number**	**Cancer Type**	**Age**	**Gender**	**Clinical Staging**	**Volume of Blood /mL**	**Captured CTCs**
1	Gastric cancer	69	Female	Ⅳ	1	8
2	Gastric cancer	77	Female	III	1	11
3	Gastric cancer	68	Female	III	1	4
4	Gastric cancer	56	Male	III	1	14
5	Gastric cancer	73	Male	III	1	7
6	Gastric cancer	61	Male	III	1	10
7	Lung cancer	59	Male	Ⅳ	1	9
8	Hepatocellular carcinoma	48	Female	Ⅳ	1	4
9	Colorectal cancer	85	Female	III	1	12
10	Hilar cholangiocarcinoma	52	Female	Ⅳ	1	9
11	Endometrial carcinoma	75	Female	III	1	6
12	Healthy	59	Male	N/A	1	0
13	Healthy	71	Female	N/A	1	0
14	Healthy	73	Male	N/A	1	0
15	Healthy	38	Female	N/A	1	0
16	Healthy	33	Male	N/A	1	0
17	Healthy	73	Male	N/A	1	0
18	Healthy	53	Female	N/A	1	0
19	Healthy	69	Female	N/A	1	0

References

1. Zhou, Y.; Wang, X.; Luo, Z.; Liu, X.; Hou, J.; Zhou, S. Efficient Isolation and In Situ Identification of Viable Circulating Tumor Cells Using Dual-Responsive Fluorescent-Magnetic Nanoparticles. *Small Sci.* **2022**, 2, 2200061.

2. He, Y.; Tian, M.; Li, X.; Hou, J.; Chen, S.; Yang, G.; Liu, X.; Zhou, S. A Hierarchical-Structured Mineralized Nanofiber Scaffold with Osteoimmunomodulatory and Osteoinductive Functions for Enhanced Alveolar Bone Regeneration. Adv. *Healthc. Mater*. **2022**, 11, 1–16.
